# Reply to Almeida, “Unreliable evidence to support a vector regulation hypothesis for *Xylella fastidiosa*–leafhopper interactions”

**DOI:** 10.1128/aem.00889-25

**Published:** 2025-07-31

**Authors:** Elaine A. Backus, Holly J. Shugart

**Affiliations:** 1 USDA-ARS San Joaquin Valley Agricultural Sciences Center 57733, Parlier, California, USA; 2 Department of Entomology, The Pennsylvania State University 8082, University Park, Pennsylvania, USA; The University of Tennessee Knoxville, Knoxville, Tennessee, USA

## REPLY

We thank Almeida for the opportunity to clarify our experimental design, evidence, and hypotheses supported. However, we disagree with Almeida’s conclusions and herein provide evidence to rebut his letter (1).

In the introduction of our 2024 paper, we state that our objective was to examine the developmental process of *Xylella fastidiosa* (*Xf*) biofilm formation on the foregut cuticle of vectors. In so doing, we proposed testing four hypotheses, only one of which (the fourth) is the vector regulation hypothesis. The first hypothesis is that there would be differences in biofilm formation between two strains of *Xf*. The second and third hypotheses are emphasized in our paper, and we explain that their support needed to precede work on the more complicated fourth hypothesis. The second hypothesis was that *Xf* biofilm formation on insect cuticle proceeds through the same stages as those on glass slides from *in vitro* studies. The third hypothesis was that the insect can *constrain* (my italics for emphasis, here and elsewhere in the rebuttal) biofilm development, via “cleaning behaviors.” Such behaviors have been studied in our earlier works and those of others (2) and definitively supported as the mechanism of *Xf* inoculation into plants (3). Thus, our third hypothesis proposed that insect behaviors could cause biofilm removal that would leave telltale marks of *disruption* of the biofilm, visible at high magnification. The fourth hypothesis, the vector *regulation* hypothesis, hinges on what we meant by “regulation.” We defined it as the ability of the insect to *detect* a threshold amount of bacterial biofilm in the foregut, which then triggers its cleaning behaviors to *regulate* the amount of biofilm present. Constraint is not the same as regulation, and we chose the word “constraint” deliberately to explain the level of evidence in our paper. A fifth hypothesis (bacterial refugia) was developed after seeing the results of the SEM study. Broadly, this series of hypotheses and the data we contribute to address them outline a homeostatic mechanism of the insect to keep the levels of biofilm in check, so they do not obstruct normal functions of the precibarial sensory organs, precibarial valve, and ultimately ingestion.

In the final paragraph of the introduction, we clearly summarize our conclusions. “Results herein support our first, second, and third hypotheses, especially the *disruption* of biofilm formation in vector foreguts due to *constraints* such as vector behavior and differences in biofilm formation between *Xf* strains. However, findings also suggest the need for additional studies to test our fourth...” hypothesis. A complicated hypothesis like the vector regulation hypothesis will no doubt require multiple, parallel but converging lines of evidence. Our paper was the second of several possible studies. It concentrated on identifying (i) stages of *Xf* biofilm development, (ii) signs of their disruption, and (iii) how that disruption revealed constraints imposed on biofilm development by insect behavior.

Regarding the reliability of our experimental design to study the three above topics, we used the same one found in many published *Xf* transmission experiments. This is a design that is familiar to all *Xf* researchers, but about which little is deeply understood. In such a classical transmission study, the insects are given an acquisition access period (AAP) on infected grapevine(s) and then are allowed to access healthy grapevine(s) for an inoculation access period (IAP). Insects may or may not be given a multiplication period on a separate plant between the AAP and IAP. In our case, we performed only the AAP part of the study. We introduced 70+ clean sharpshooter vectors into a cage with *Xf*-infected grapevines and then removed 10 insects per day for 7 AAP days, for testing. The first time we used this design was for a confocal microscopy study (4) when we asked the question “What is the distribution of bacterial biofilm in various parts of the complicated foregut anatomy for each AAP day?” The second time was for the PCR study in the paper in question, when we asked, “How many bacteria are present in a whole foregut/head for each AAP day?” Results of the confocal study surprisingly revealed that biofilm amount and locations apparently increased and decreased across the 7 AAP days, a finding later tested and supported by our PCR study herein.

We chose a 7-day timespan for our studies to include all presently used AAPs as a bridge to numerous past studies. Multiple AAPs have been used in papers studying *Xf* transmission over the last 80+ years. A survey of the literature in the last 20 years found 18 papers (some in just the last 5 years) that used diverse AAPs with no intervening multiplication period before the inoculation access period (IAP) was applied. Six of these 18 papers were authored or co-authored by Almeida. Most AAP studies use a single day, such as 2 days (5–9) or 4 days (10–14), or a range of days, such as 1–2 days (15), 1–5 days (15), or 2–3 days (16). To keep the interpretation of our study simple, we chose not to use a multiplication period after our AAP, although some studies use one (17).

Classical transmission studies were originally designed decades before recent findings that *Xf* biofilm formation proceeds through five distinct developmental stages whose criteria are adhesive types and bacterial morphology, visible only using high-magnification, scanning electron microscopyy (SEM). *Xf* biofilm is mostly mature by 8 days *in vitro* (18, 19); evidence from plant and insect tissues supports similar timeframes on living substrates like plant and insect tissues (20). Once we learned about such developmental stages, we sought to use SEM to investigate such stages. However, the classical design was changed slightly for our SEM study because of the technical difficulty of head dissections for such work, allowing time for only 1 AAP day to be used. Based on the earlier study (4), we chose AAP day 4 because biofilm was being expelled by then. Thus, the SEM study asked the question “What is the appearance of adhesives and bacterial morphology of these biofilm stages at one AAP day?”

Our SEM results were of the highest quality and resolution possible today, with modern critical point drying methods that preserved the many and varied adhesive layers, unlike most previous SEM examinations of *X. fastidiosa* biofilm on insect cuticle. This was the key to our findings about stages of *Xf* biofilm development and their disruption. In our text section on “Mechanical removal of adhesive layers from the cuticle,” we stated that “Direct observation of adhesive layers on the cuticle also allowed us to see extreme degradation of the layers, including tearing and stripping of whole swaths of material, rolling, curling, and various displacements of material, leading to reductions and removal of layers that revealed underlying, thinner layers. No evidence from *in vitro* or *in planta* studies shows such physical disruption of EPS during post-maturation degradation of mature biofilm; rather, visual evidence up to 8 d suggests that adhesive accumulates for subsequent generations of cells to move through… Thus, the degraded appearance of EPS adhesives is unique to insect cuticle, suggesting that actions by the insect… physically damaged and often scrubbed off the EPS layers and their associated micro-colonies from…” the foregut.

Many more details and discussion can be found in our paper, but the section “Conclusions about our hypotheses” is most relevant to our rebuttal. “When combined with results from fluid hydrodynamic studies of the vector foregut during ingestion and egestion…, the proven inoculation mechanism for *Xf*, and results from our head PCR study, our SEM results support our Hypotheses 2 and 3, especially that insect vectors can *constrain* development of typical *Xf* biofilm stages on their foregut cuticles by actively removing/dispersing existing bacteria, separably from bacterial self-dispersal. In particular, our evidence supports that the insect is capable of mechanically scrubbing off reversibly attached S-EPS adhesive layers, and likely irreversibly attached layers also.” These unequivocal results, in the context of a well-known and used experimental design, show that while *Xf* biofilm development proceeds through the same stages, the process is interrupted in insects compared with uninterrupted development on glass slides.

It was apparently not clear to Almeida that we were *proposing* the vector regulation hypothesis (4) in our paper’s title but then explaining in the same title support for hypothesis 3 (*constraint*), a critical step on the way towards definitively supporting vector regulation. Furthermore, the distinction between *constraint* (hypothesis 3) and *regulation* (hypothesis 4) was clearly stated in our abstract: “Evidence supports the hypothesis that bacterial colonization was repeatedly interrupted and *constrained* by the vector. Behaviors such as egestion and enzymatic salivation likely can loosen and eject *Xf* biofilm [respectively], perhaps when profuse biofilm interferes with ingestion. Thus, vector acquisition of *Xf* is a dynamic and stochastic process of interactions between bacteria and insects. We further hypothesize for *future* testing that the insect can *regulate* this interaction.”

The definition of “regulation” in the context of *X. fastidiosa* biofilm is further discussed in our section on “Conclusions about our hypotheses,” as follows: “*Additional tests* will be required for evidence of prevention of biofilm development... [thus] that the insect *regulates Xf* biofilm formation when it exceeds a threshold volume that could interfere with ingestion...” We clearly explained that, while hypotheses 2 and 3 laid critical groundwork, they could not provide direct support for hypothesis 4.

Next, we stated that definitive evidence would be provided by a future study very similar to the one proposed by Almeida in his letter. Following the above quote: “An excellent future test could involve a 7-d (or more) time course.” We wrote this conclusion because we recognized, precisely as Almeida contends that (quote from his letter) “by continuously exposing insects to infected plants over 7 days, it is impossible to determine (i) when pathogen acquisition occurred and (ii) how many times pathogen acquisition occurred.” While we agree with Almeida that continuous exposure could not directly test hypothesis 4, continuous exposure was necessary and critical to bridge between the common and accepted design of a classical transmission study and entirely new, future work based on biofilm structure and stages. Also, it will be a challenge to identify a useful AAP for a pulse-chase, time course study in the future. While Almeida’s design is interesting and useful, much thought will need to be given to identify the best one or two designs for that future study.

In summary, our experimental design for the PCR study followed the same design as many *Xf* transmission studies preceding ours. It was not flawed, nor was the preceding paper (4) on which this one was based. Both studies provided valuable information that was not researched before, but their results need to be considered in the context of the hypotheses and questions asked. Our experimental design for the SEM study was similarly flawless. The interpretation of our experiments, and whether they definitively support the vector regulation hypothesis, was clearly stated in both the introduction and the conclusions of our paper. They supported the hypothesis for *constraint* of biofilm formation, but not for *regulation*.

We uncategorically refute Almeida’s claim that our evidence was “unreliable”; it was extremely reliable and high-quality evidence for the hypotheses that were tested, hypotheses 2 and 3, but not (as we admitted in the paper) for hypothesis 4, the vector regulation hypothesis. A complicated hypothesis like the vector regulation hypothesis will no doubt require multiple, parallel but converging lines of evidence over time, just as did the *Xf* inoculation hypothesis that egestion was the mechanism. We were entirely honest in our 2024 title, abstract, and the rest of the paper. It seems Almeida did not understand our distinction between *constraint* and *regulation*. Science is a process, and that process does not end with the introduction of a new hypothesis. Rather, hypotheses are tested and often adapted based on newly contributed data. We welcome other researchers, including Almeida, to join in the pursuit of testing our vector regulation hypothesis in future studies.
